# Cerebrospinal Fluid p75 Neurotrophin Receptor Ectodomain Levels Across Clinically Diagnosed Alzheimer's Disease, Late‐Life Depression, and Healthy Aging: A Preliminary Cross‐Sectional Study

**DOI:** 10.1002/npr2.70171

**Published:** 2026-09-20

**Authors:** Wataru Omori, Naoto Kajitani, Mami Okada‐Tsuchioka, Minoru Takebayashi

**Affiliations:** ^1^ Department of Psychiatry NHO Kure Medical Center and Chugoku Cancer Center Kure Hiroshima Japan; ^2^ Division of Psychiatry and Neuroscience, Institute for Clinical Research NHO Kure Medical Center and Chugoku Cancer Center Kure Hiroshima Japan; ^3^ Department of Psychiatry and Neuroscience, Center for Metabolic Regulation of Healthy Aging, Faculty of Life Science Kumamoto University Kumamoto Japan

**Keywords:** Alzheimer's disease, cerebrospinal fluid, healthy aging, late‐life depression, p75 neurotrophin receptor ectodomain

## Abstract

**Objective:**

The p75 neurotrophin receptor ectodomain (p75NTR‐ECD) has been implicated in amyloid‐β‐related neurotoxicity, but cerebrospinal fluid (CSF) levels in late‐life depression (LLD) remain uncertain. We compared clinically diagnosed Alzheimer's disease (AD), LLD, and healthy controls (HC) and reassessed exploratory symptom and cognitive associations.

**Methods:**

CSF p75NTR‐ECD was measured in 50 participants (HC, *n* = 19; LLD, *n* = 19; AD, *n* = 12). The primary linear model adjusted for age, sex, and body mass index. Exploratory symptom and cognitive models additionally adjusted for diagnostic group; six *p* values underwent Benjamini–Hochberg false discovery rate (FDR) correction. Plate and below‐calibrator sensitivity analyses were performed.

**Results:**

CSF p75NTR‐ECD was higher in HC than in AD (B = 131.2 pg/mL, 95% CI 42.8–219.6, *p* = 0.005) and higher in LLD than in AD (B = 154.6 pg/mL, 95% CI 68.9–240.3, *p* < 0.001); HC and LLD did not differ. No symptom or cognitive association remained significant after diagnostic‐group adjustment and FDR correction (all q ≥ 0.117). Plate adjustment did not materially change the primary contrasts. Excluding three concentrations below the lowest calibrator preserved the LLD–AD contrast but attenuated the HC–AD contrast.

**Conclusions:**

CSF p75NTR‐ECD showed preliminary differences across clinically defined groups. Because the sample was small, the recruitment site was perfectly confounded with AD versus non‐AD status, and diagnoses were not uniformly biomarker‐confirmed, the findings do not establish causality, clinical diagnostic utility, or added value beyond established AD biomarkers and require independent multisite replication.

## Abstract 2

CSF p75NTR‐ECD was lower in clinically diagnosed Alzheimer's disease than in late‐life depression or healthy aging in the primary covariate‐adjusted analysis. Exploratory symptom and cognitive associations did not survive diagnostic‐group adjustment and FDR correction. Site–diagnosis confounding requires cautious interpretation and independent multisite replication.

## Graphical abstract

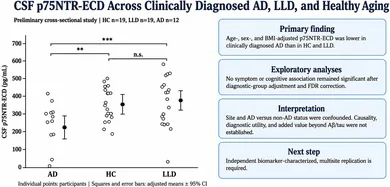

## 1 Introduction

Differentiating late‐life depression (LLD) from Alzheimer's disease (AD) can be difficult because affective and cognitive symptoms frequently overlap. Established cerebrospinal fluid (CSF) amyloid‐β and tau biomarkers can support an AD diagnosis, but biologically heterogeneous LLD cohorts may include both amyloid‐negative and amyloid‐positive individuals [1, 2, 3, 4, 5, 6, 7, 8, 9]. Additional mechanism‐linked measures may therefore be useful for hypothesis generation, although their value must be assessed against established biomarker frameworks.

Brain‐derived neurotrophic factor (BDNF) is synthesized as proBDNF and processed into mature BDNF and BDNF pro‐peptide, which have distinct biological actions [10, 11, 12]. Whereas mature BDNF preferentially signals through TrkB, proBDNF can engage p75NTR and facilitate hippocampal long‐term depression [13]. BDNF pro‐peptide can also facilitate hippocampal long‐term depression [14]. Alterations in BDNF‐related measures have been reported in major depressive disorder, suicide, and antidepressant‐treated tissue or serum [15, 16, 17, 18, 19, 20]. p75NTR has also been implicated in amyloid‐β‐related neurotoxicity [21, 22]. Its soluble ectodomain (p75NTR‐ECD), generated by ADAM17/TACE‐mediated shedding, can bind amyloid‐β and has shown neuroprotective properties in experimental models [21, 23, 24].

Prior work reported lower CSF p75NTR‐ECD in AD and an association with global cognition [25]. However, CSF p75NTR‐ECD has not been systematically characterized in LLD, and earlier findings do not establish clinical diagnostic utility or added value beyond amyloid and tau biomarkers. Symptom‐ and cognition‐related associations may also be confounded by diagnostic group because AD, LLD, and healthy aging differ substantially in their clinical profiles.

The primary aim of this preliminary cross‐sectional study was to compare CSF p75NTR‐ECD levels across clinically diagnosed AD, LLD, and healthy control (HC) groups while adjusting for age, sex, and body mass index (BMI). Secondary analyses examined depressive‐symptom and cognitive measures after additional adjustment for diagnostic group and false discovery rate control. The study was not designed to establish causality, a clinical cutoff, or incremental value beyond established AD biomarkers.

## 2 Methods

### 2.1 Participants

We enrolled Japanese participants from two sites: patients with LLD from the National Hospital Organization Kure Medical Center (NHOKMC), Hiroshima (January 2015–May 2018), patients with AD from Kumamoto University, Kumamoto (September 2009–July 2017), and HC recruited at NHOKMC through an announcement on the Newing NPO Corporation website (September 2017–May 2019). All individuals underwent a structured diagnostic interview using the Mini‐International Neuropsychiatric Interview [26] (Japanese version, MINI‐J [27]), administered by trained clinical psychologists or psychiatrists. In participants aged ≥ 50 years with major depressive disorder or AD, a consensus diagnosis according to DSM‐IV‐TR criteria [28] was established using the MINI‐J, additional clinical interviews, and medical‐record review.

The study included 19 participants with LLD, 12 with clinically diagnosed AD, and 19 HC without a past or current mental disorder. Individuals with a history of central nervous system disease other than AD, severe head injury, or substance abuse were excluded. AD diagnosis was clinically based. Legacy AD‐related biomarker values are included in Supporting Information 1 where available; however, no uniform biomarker protocol was used across groups, amyloid‐β42 values were available only in the AD group, and amyloid‐β42/40 ratios and total tau were unavailable. Biomarker‐supported differential diagnosis and analyses of independence or added value relative to core AD biomarkers were therefore not possible.

Recruitment site was perfectly confounded with AD versus non‐AD status: all AD participants were recruited at Kumamoto University, whereas all LLD and HC participants were recruited at NHOKMC. Site and diagnostic‐group effects could therefore not be estimated simultaneously.

The study complied with the Declaration of Helsinki and was approved by the ethics committees of NHOKMC and Kumamoto University. All participants provided written informed consent.

### 2.2 Clinical Assessments

Depressive symptoms were assessed with the 21‐item Hamilton Rating Scale for Depression (HAM‐D‐21), and global cognition was assessed with the Mini‐Mental State Examination (MMSE). Exploratory domains were defined from prespecified item groupings available in the dataset: HAM‐D‐21 sleep (items 4–6), psychic anxiety (items 9–10), MMSE orientation (items 1–2), and MMSE delayed recall (item 5).

### 2.3 Medication Status

Current medications were systematically reviewed. Antidepressant doses were converted to imipramine (IMI) equivalents and antipsychotic doses to chlorpromazine (CPZ) equivalents using Japanese dose‐equivalence tables [29]. Benzodiazepine receptor agonists were harmonized to approximate diazepam milligram equivalents (DME) [29].

All LLD participants received antidepressants; some also received antipsychotic augmentation, benzodiazepines, or hypnotics. No AD participant received an antidepressant or antidementia medication; two received antipsychotics, two received benzodiazepines, and three received hypnotic agents. Because dose equivalence does not capture class‐specific pharmacodynamics, medication‐related residual confounding remained possible.

### 2.4 Lumbar Puncture and CSF Handling

Lumbar puncture was performed at the L3–L4 or L4–L5 level according to previously described procedures [30]. Eight milliliters of CSF were collected into low‐protein‐adsorption polypropylene tubes and immediately chilled on ice. CSF was centrifuged at 4000 g for 10 min at 4°C; supernatants were aliquoted into 0.5‐mL low‐protein‐adsorption tubes and stored at −80°C until assay. Storage duration before assay ranged from 1.3 to 5.7 years. All AD and HC samples underwent one freeze–thaw cycle; ten LLD samples underwent two cycles.

### 2.5 Measurement of CSF p75NTR‐ECD

All p75NTR‐ECD measurements were performed on September 18, 2020, in a research laboratory within the Department of Psychiatry at Kumamoto University using a human NGFR/p75 ECD enzyme‐linked immunosorbent assay kit (Biosensis, South Australia) and a Multiskan GO microplate spectrophotometer (Thermo Fisher Scientific, Waltham, MA, USA). Undiluted CSF was measured in duplicate on three plates during the same assay session. All plates used kits from the same manufacturing lot.

HC, LLD, and AD samples were distributed approximately evenly across the three plates, and every plate contained samples from all three groups (plate 1: 7 HC, 7 LLD, and 4 ad; plates 2 and 3: 6 HC, 6 LLD, and 4 ad each). Well placement was not randomized. Samples were identified by codes, and the assay operator was unaware of the diagnostic group.

The standard curve ranged from 62.5 to 4000 pg/mL and was fitted using a four‐parameter logistic model. Three concentrations (one LLD and two AD) were below the lowest calibrator; the assay software estimated these values by extrapolating the fitted curve toward the lower‐concentration range. No prespecified repeat‐assay criterion was used, and samples were not re‐assayed. The median duplicate coefficient of variation was 2.4% (range, 0.1%–19.3%). Because all measurements were performed in one session and each sample was assigned to one plate, independent inter‐assay precision could not be estimated.

### 2.6 Statistical Analysis

Continuous variables were summarized as mean ± standard deviation. Normality was assessed using the Shapiro–Wilk test. Group comparisons used Kruskal–Wallis tests with Dunn–Bonferroni post hoc comparisons where applicable; categorical variables used chi‐square tests. Medication‐equivalent variables were compared between LLD and AD using two‐sided Mann–Whitney U tests because HC values were structural zeros.

The primary analysis modeled CSF p75NTR‐ECD as the dependent variable, with diagnostic group as the main explanatory variable and AD as the reference, adjusting for age, sex, and BMI. The diagnostic‐group contribution was evaluated with a two‐degree‐of‐freedom partial F test. Adjusted marginal means were estimated at the sample means of age, BMI, and sex. HC3 heteroscedasticity‐consistent standard errors were examined as a sensitivity analysis. Residual normality, heteroscedasticity, independence, and multicollinearity were assessed using Q–Q plots, the Shapiro–Wilk test, Breusch–Pagan and White tests, the Durbin–Watson statistic, and variance inflation factors.

For each of six exploratory symptom or cognitive predictors, a separate linear model included that predictor, age, sex, BMI, and diagnostic group. Nominal *p* values for the six predictor coefficients were adjusted using the Benjamini–Hochberg false discovery rate (FDR) procedure. HC3‐based results were also tabulated. Three exploratory medication‐dose models within LLD were treated as a separate family and were likewise FDR adjusted.

Assay‐related sensitivity analyses additionally adjusted the primary model for plate, storage duration, or freeze–thaw cycles in separate models. To examine dependence on the three software‐estimated concentrations below the lowest calibrator, these values were either set to 62.5 pg/mL or excluded.

Two‐sided *p* < 0.05 was used for nominal inference; FDR‐adjusted *q* < 0.05 defined significance for exploratory families. Analyses were performed in Python 3.13 using statsmodels 0.14.6 and SciPy 1.17.0.

During revision, OpenAI ChatGPT (GPT‐5.6 Pro; accessed August 2026) was used to assist with drafting and checking statistical code. All model specifications, code, numerical outputs, and interpretations were reviewed and verified by the authors against the coded source dataset. The tool did not generate, alter, or impute any original research data.

## 3 Results

### 3.1 Participant Characteristics

The study included 50 participants (HC, *n* = 19; LLD, *n* = 19; AD, *n* = 12), comprising 31 men and 19 women; mean age was 66.5 ± 10.0 years. Sex distribution did not differ across groups (*p* = 0.765), whereas age (*p* = 0.005), BMI (*p* = 0.003), HAM‐D‐21 score (*p* < 0.001), and MMSE score (*p* < 0.001) did (Table 1). IMI equivalents were higher in LLD than AD (*p* < 0.001), CPZ equivalents did not differ (*p* = 0.251), and DME was higher in LLD (*p* = 0.049).

**TABLE 1:** Participant characteristics and clinical measures.

	HC (*n* = 19)	LLD (*n* = 19)	AD (*n* = 12)	*p*
Sex, female	6 (31.6%)	8 (42.1%)	5 (41.7%)	0.765^a^
Age (y)	61.5 ± 8.4	72.0 ± 8.9	65.6 ± 10.0	0.005^b^ ^,^ **
BMI (kg/m^2^)	22.9 ± 2.2	20.4 ± 3.7	20.3 ± 1.4	0.003^b^ ^,^ **
HAM‐D‐21	2.0 ± 1.6	10.6 ± 7.0	6.9 ± 4.3	< 0.001^b^ ^,^ ***
MMSE	27.5 ± 1.9	26.7 ± 1.8	17.3 ± 8.2	< 0.001^b^ ^,^ ***
IMI equivalents (mg/day)	0	236.8 ± 130.3	0	< 0.001^c^ ^,^ ***
CPZ equivalents (mg/day)	0	36.4 ± 71.4	56.3 ± 172.6	0.251^c^
DME (mg/day)	0	5.0 ± 5.9	0.8 ± 1.9	0.049^c^ ^,^ *

*Note:* Data are presented as mean ± standard deviation unless otherwise indicated; categorical variables are *n* (%).

Abbreviations: AD, Alzheimer's disease; BMI, body mass index; CPZ, chlorpromazine; DME, diazepam milligram equivalents; HAM‐D‐21, 21‐item Hamilton Rating Scale for Depression; HC, healthy controls; IMI, imipramine; LLD, late‐life depression; MMSE, Mini‐Mental State Examination.

^a^ Three‐group chi‐square test.

^b^ Global Kruskal–Wallis test.

^c^ Two‐sided Mann–Whitney U test comparing LLD with AD for medication‐equivalent variables; HC values were structural zeros.

* *p* < 0.05.

** *p* < 0.01.

*** *p* < 0.001.

### 3.2 Primary Between‐Group Analysis

**FIGURE 1:**
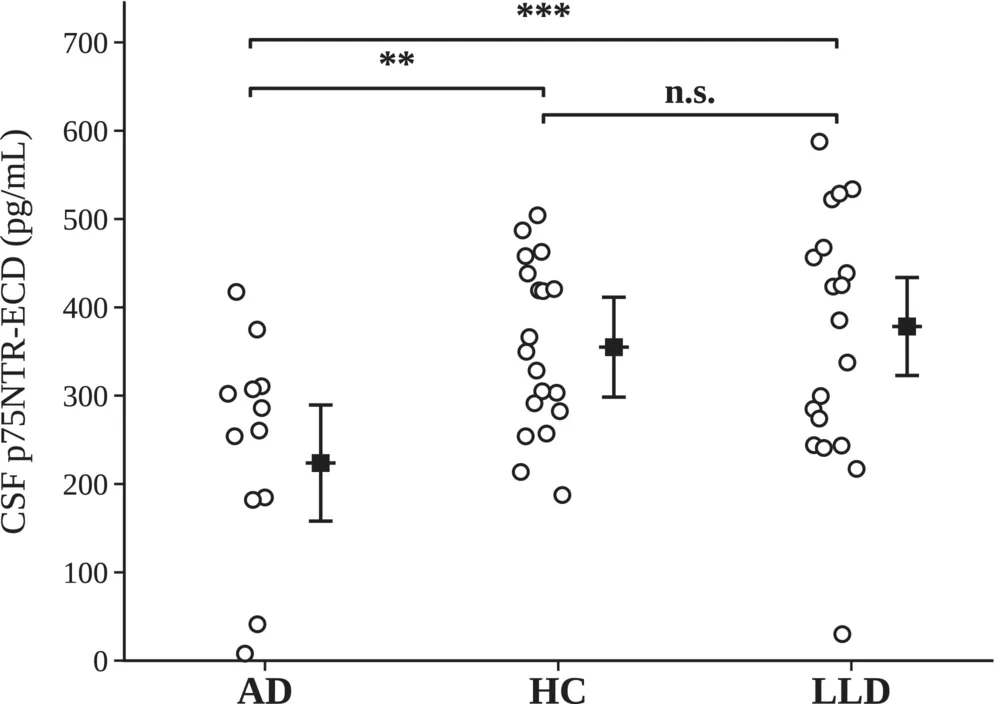
Individual CSF p75NTR‐ECD concentrations across clinically defined diagnostic groups. Open circles represent individual participants. Black squares and error bars indicate age‐, sex‐, and BMI‐adjusted marginal means with 95% confidence intervals from the primary model: AD, 223.7 pg/mL (95% CI 157.9–289.5); HC, 354.9 pg/mL (298.4–411.5); and LLD, 378.3 pg/mL (322.8–433.8). Pairwise contrasts were AD versus HC, *p* = 0.005; AD versus LLD, *p* < 0.001; and HC versus LLD, *p* = 0.583. The comparison is preliminary because recruitment site was confounded with AD versus non‐AD status. Abbreviations: AD, Alzheimer's disease; BMI, body mass index; CSF, cerebrospinal fluid; HC, healthy controls; LLD, late‐life depression; p75NTR‐ECD, p75 neurotrophin receptor ectodomain. **p < 0.01; ***p < 0.001; n.s., not significant.

In the primary model, diagnostic group contributed significantly after adjustment for age, sex, and BMI (partial F‐test *p* = 0.002). Compared with AD, CSF p75NTR‐ECD was higher in HC (B = 131.2 pg/mL, 95% CI 42.8–219.6, *p* = 0.005) and LLD (B = 154.6 pg/mL, 95% CI 68.9–240.3, *p* < 0.001); HC and LLD did not differ (B = −23.4 pg/mL, 95% CI −108.5 to 61.7, *p* = 0.583). Age and BMI were inversely associated with p75NTR‐ECD, whereas sex was not. The model explained 33.7% of variance (adjusted R^2^ = 0.261; Table 2). Adjusted marginal means were 223.7 pg/mL for AD, 354.9 pg/mL for HC, and 378.3 pg/mL for LLD (Figure 1).

**TABLE 2:** Multivariable linear regression for CSF p75NTR‐ECD.

Variable	B	95% CI	Standardized β	*p*
Constant	905.713	518.130 to 1293.296	—	< 0.001***
Age	−5.138	−8.838 to −1.439	−0.397	0.008**
Sex (male)	−21.673	−87.242 to 43.896	−0.082	0.509
BMI	−15.335	−27.461 to −3.210	−0.352	0.014*
HC vs. AD	131.208	42.831 to 219.585	0.499	0.005**
LLD vs. AD	154.586	68.901 to 240.272	0.588	< 0.001***

*Note: R* ^2^ = 0.337; adjusted *R* ^2^ = 0.261; overall model *p* = 0.002. Diagnostic‐group partial F‐test *p* = 0.002.

Abbreviations: AD, Alzheimer's disease; BMI, body mass index; CI, confidence interval; CSF, cerebrospinal fluid; HC, healthy controls; LLD, late‐life depression.

* *p* < 0.05.

** *p* < 0.01.

*** *p* < 0.001.

### 3.3 Exploratory Symptom and Cognitive Analyses

After additional adjustment for diagnostic group, nominal associations were observed for the HAM‐D‐21 psychic anxiety domain (B = −37.1, *p* = 0.048) and MMSE delayed recall (B = 47.9, *p* = 0.027). However, none of the six symptom or cognitive associations remained significant after FDR correction (all q ≥ 0.117; Table S1). The remaining nominal *p* values ranged from 0.058 to 0.610. Thus, these analyses did not provide robust evidence of symptom‐ or cognition‐specific associations independent of diagnostic group.

### 3.4 Assay‐Related Sensitivity Analyses

Adding plate to the primary model did not materially change the contrasts (HC vs. AD: B = 133.4 pg/mL, *p* = 0.003; LLD vs. AD: B = 151.0 pg/mL, *p* < 0.001). Setting the three values below the lowest calibrator to 62.5 pg/mL also preserved both contrasts. When the three values were excluded (*n* = 47), the LLD–AD contrast remained significant (B = 124.5 pg/mL, 95% CI 44.8–204.2, *p* = 0.003), whereas the HC–AD contrast was attenuated (B = 78.3 pg/mL, 95% CI −2.8 to 159.5, *p* = 0.058; Table S2).

### 3.5 Exploratory Medication Analyses

Within LLD, p75NTR‐ECD was not associated with IMI equivalents, CPZ equivalents, or DME; all medication‐family FDR q values were 0.895 (Table S3 and Figure S1). These analyses do not exclude class‐specific medication effects.

### 3.6 Assay Quality and Model Diagnostics

All three diagnostic groups were represented on each of the three assay plates, and the median duplicate coefficient of variation was 2.4% (range, 0.1%–19.3%; Table S4). Model diagnostics did not indicate major assumption violations (Shapiro–Wilk *p* = 0.146; Breusch–Pagan *p* = 0.501; White *p* = 0.272; Durbin–Watson = 1.854), and all variance inflation factors were below 1.9. The corresponding Q–Q plot is shown in Figure S2. HC3 standard errors did not materially alter the primary contrasts.

## 4 Discussion

### 4.1 Principal Findings

In this small cross‐sectional cohort, CSF p75NTR‐ECD differed across clinically defined diagnostic groups after adjustment for age, sex, and BMI. The AD group had lower levels than the LLD and HC groups in the primary analysis, whereas LLD and HC did not differ. The LLD–AD contrast was consistent across plate and below‐calibrator sensitivity analyses; the HC–AD contrast was less robust when the three values below the lowest calibrator were excluded.

### 4.2 Biological and Clinical Interpretation

The direction of the group difference is consistent with prior work reporting lower CSF p75NTR‐ECD in AD [25]. p75NTR‐ECD is generated through ADAM17/TACE‐mediated shedding and can bind amyloid‐β in experimental systems, providing a plausible biological context [21, 23, 24]. Amyloid‐β1–42 has also been shown to induce neuronal death through p75NTR in an experimental model [31]. However, the present data do not measure shedding activity, amyloid binding, mature BDNF, proBDNF, BDNF pro‐peptide, or TACE activity. The biological explanation therefore remains hypothetical rather than demonstrated.

The revised exploratory analyses materially changed interpretation. Associations previously observed without diagnostic‐group adjustment were reduced after the diagnostic group was included, and none survived FDR correction. Although psychic anxiety and delayed recall had nominal *p* values below 0.05, their q values were 0.117. Related BDNF‐family measures have been associated with cognition or AD‐related pathology in prior studies [32, 33, 34], but these analytes are biologically and analytically distinct from CSF p75NTR‐ECD. The current results do not establish independent symptom‐domain associations. These findings are best viewed as descriptive and should not be used to infer symptom‐specific mechanisms.

Medication exposure also complicates interpretation. All LLD participants received antidepressants, and subsets received antipsychotics, benzodiazepines, or hypnotics. Although dose‐equivalent measures were not linearly associated with p75NTR‐ECD, dose equivalence cannot capture class‐specific pharmacodynamics. Experimental and clinical literature suggests that serotonergic treatment may influence components of the BDNF/p75NTR pathway [35, 36, 37], so residual medication confounding cannot be excluded.

The study was not designed to evaluate clinical diagnostic utility or derive diagnostic thresholds, and no external validation was available. Furthermore, the absence of uniform amyloid and tau characterization precludes assessment of whether p75NTR‐ECD provides information independent of or additive to established AD biomarkers. Its potential role should therefore be tested only in biomarker‐characterized, independently recruited multisite cohorts.

### 4.3 Limitations

Several limitations are important. First, the sample was small, particularly the AD group, limiting precision, stability, and generalizability. Second, the design was cross‐sectional and cannot establish temporal sequence, causality, prediction, or longitudinal change. Third, AD was clinically diagnosed without uniform biomarker confirmation, creating potential misclassification. Vascular comorbidity, inflammatory status, APOE genotype, and detailed disease severity were not uniformly available and could not be incorporated without substantial overfitting.

Fourth, the recruitment site was perfectly confounded with AD versus non‐AD status. Consequently, the observed AD–non‐AD differences cannot be attributed solely to diagnosis and may partly reflect site, cohort, referral, storage duration, or other preanalytical differences. Plate was not confounded with group and plate adjustment did not materially change estimates, but this does not resolve the site confounding. Fifth, ten LLD samples underwent an additional freeze–thaw cycle, and storage duration differed across cohorts.

Sixth, well placement was not randomized, no prespecified repeat‐assay criterion was used, and independent inter‐assay precision could not be estimated. Three concentrations were extrapolated below the lowest calibrator; the sensitivity analyses showed that the HC–AD comparison was partly dependent on their treatment. Seventh, the LLD cohort was clinically heterogeneous, and cognition was assessed only with the MMSE, which has ceiling effects and limited domain sensitivity. Finally, although FDR correction reduced false‐positive risk, the exploratory analyses remained underpowered and require independent confirmation.

### 4.4 Conclusions

CSF p75NTR‐ECD showed a preliminary between‐group difference in this small clinically defined cohort, with lower levels in AD in the primary covariate‐adjusted analysis. The LLD–AD contrast was the more consistent comparison across assay‐related sensitivity analyses. These observations do not establish causality, clinical diagnostic utility, or added value beyond established AD biomarkers. Replication in larger, biomarker‐characterized, multisite cohorts with standardized preanalytical procedures is required.

## Author Contributions

W.O. designed the study; W.O. and M.T. recruited participants and acquired the clinical data; N.K. performed the ELISA assay; W.O. performed the statistical analyses; M.T. supervised the study. All authors interpreted the data, critically revised the manuscript, and approved the final version.

## Funding

This work was supported by JSPSKAKENHI Grant numbers 23K14818 to W.O., 18H02756 to M.T., and 18K07620 to M.O.‐T.

## Conflicts of Interest

The authors declare no conflicts of interest.

## Supporting information


**Figure S1:** Associations between CSF p75NTR‐ECD and psychotropic dose‐equivalent measures within LLD. Crosses indicate individual participants. Lines show univariable least‐squares fits for (A) IMI equivalents, (B) CPZ equivalents, and (C) DME. Nominal *p* values are displayed in the panels; the Benjamini–Hochberg FDR q value was 0.895 for each analysis. Abbreviations: CPZ, chlorpromazine; CSF, cerebrospinal fluid; DME, diazepam milligram equivalents; FDR, false discovery rate; IMI, imipramine; LLD, late‐life depression; p75NTR‐ECD, p75 neurotrophin receptor ectodomain.

**Figure S2:** Q–Q plot of standardized residuals from the primary linear model. Open circles indicate standardized residuals, and the line indicates the theoretical normal reference. Residuals were approximately normal (Shapiro–Wilk W = 0.965, *p* = 0.146). Breusch–Pagan *p* = 0.501, White *p* = 0.272, and Durbin–Watson = 1.854 provided no evidence of major heteroscedasticity or autocorrelation.

**Table S1:** Diagnostic‐group‐adjusted symptom and cognitive analyses.
**Table S2:** Sensitivity analyses for the primary group contrasts.
**Table S3:** Exploratory medication‐dose analyses within LLD.
**Table S4:** Assay plate allocation and duplicate precision.

**Supporting Information 1:** De‐identified participant‐level dataset and analysis outputs. The workbook includes de‐identified variables used in the primary and exploratory analyses, a data dictionary, data‐quality checks, assay plate and duplicate information, Table 1 recalculation, primary and sensitivity‐model results, FDR‐adjusted exploratory analyses, and analysis metadata. Direct identifiers, exact CSF collection dates, and free‐text clinical notes were removed.

## Data Availability

The de‐identified participant‐level data that support the findings of this study are available in Supporting Information 1, together with a data dictionary, assay‐quality variables, and analysis outputs. Direct identifiers, exact CSF collection dates, and free‐text clinical notes have been removed. Additional information that could increase re‐identification risk is not publicly shared.
