# Supplementary material for: Cerebrospinal Fluid p75 Neurotrophin Receptor Ectodomain Levels Across Clinically Diagnosed Alzheimer's Disease, Late‐Life Depression, and Healthy Aging: A Preliminary Cross‐Sectional Study
Source: Neuropsychopharmacol Rep. 2026 Sep 20;46(3):e70171. doi: 10.1002/npr2.70171 (PMC13590209; doi:10.1002/npr2.70171)

Supplementary Figure S1

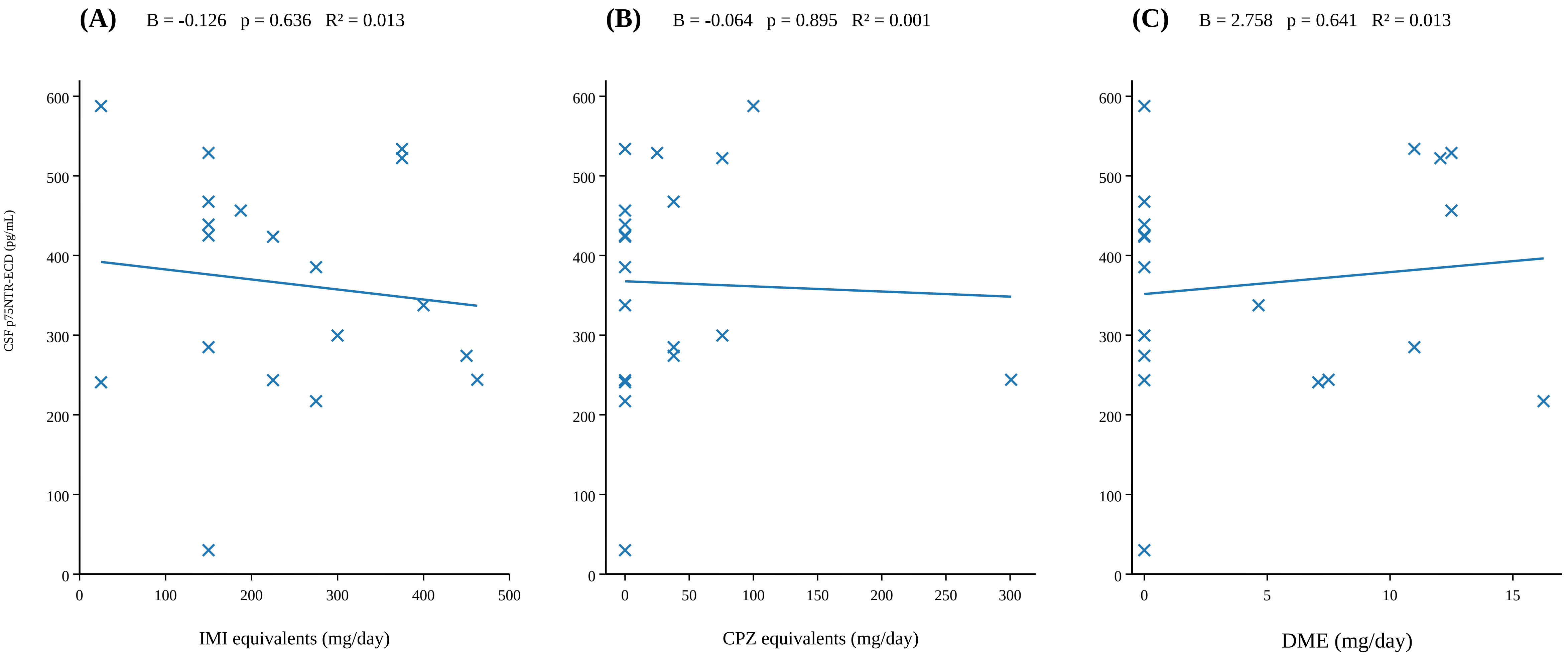

Supplement: Supplementary file 1 [file NPR2-46-e70171-s002.pdf]
