# Supplementary material for: Cerebrospinal Fluid p75 Neurotrophin Receptor Ectodomain Levels Across Clinically Diagnosed Alzheimer's Disease, Late‐Life Depression, and Healthy Aging: A Preliminary Cross‐Sectional Study
Source: Neuropsychopharmacol Rep. 2026 Sep 20;46(3):e70171. doi: 10.1002/npr2.70171 (PMC13590209; doi:10.1002/npr2.70171)

Supplementary Figure S2

Q-Q Plot of Residuals (Primary Model)

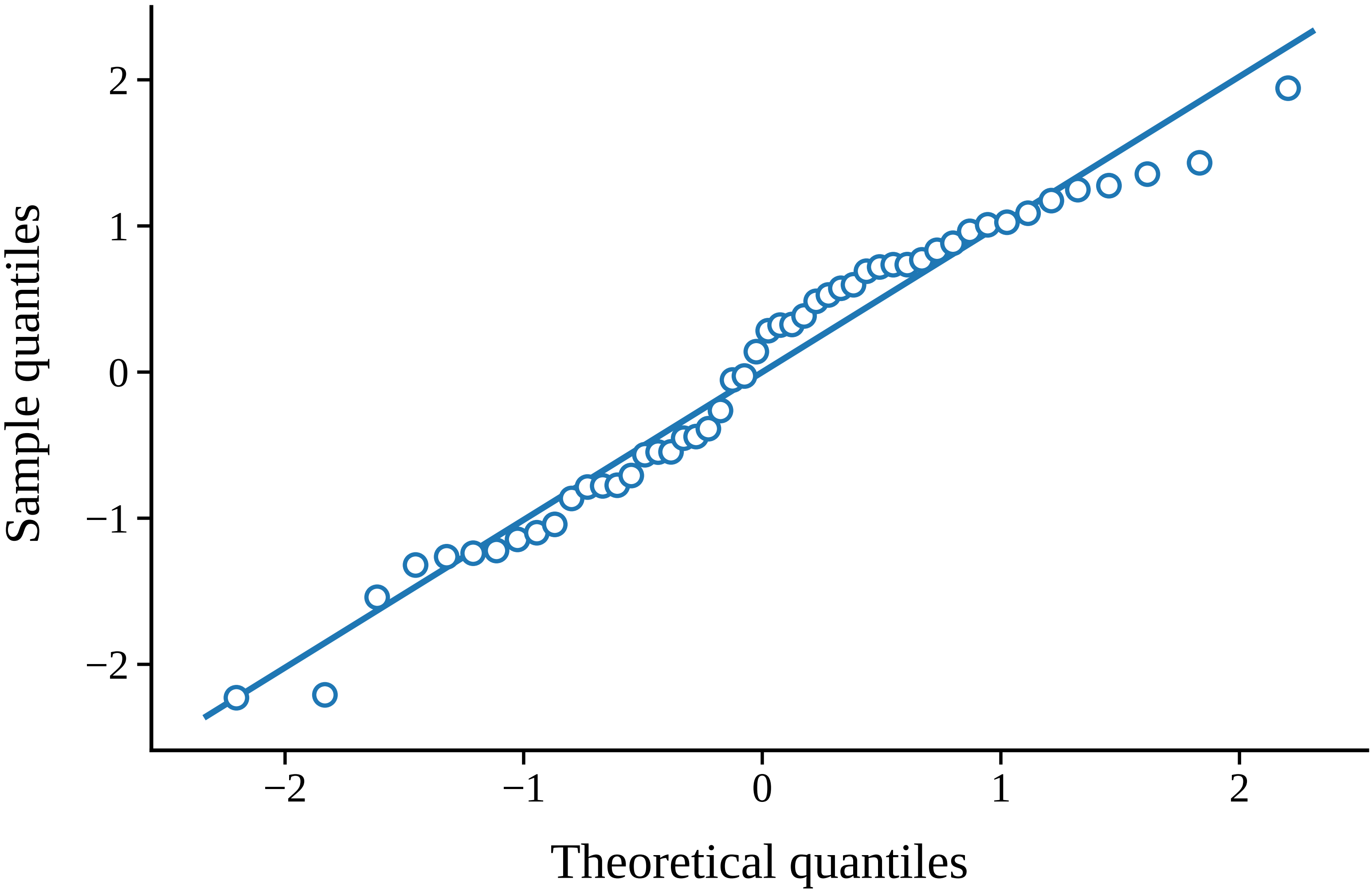

Supplement: Supplementary file 2 [file NPR2-46-e70171-s003.pdf]
