# Supplementary material for: Cerebrospinal Fluid p75 Neurotrophin Receptor Ectodomain Levels Across Clinically Diagnosed Alzheimer's Disease, Late‐Life Depression, and Healthy Aging: A Preliminary Cross‐Sectional Study
Source: Neuropsychopharmacol Rep. 2026 Sep 20;46(3):e70171. doi: 10.1002/npr2.70171 (PMC13590209; doi:10.1002/npr2.70171)
Supplement: Supplementary file 3 [file NPR2-46-e70171-s001.docx]

# Supplementary Material

Cerebrospinal Fluid p75 Neurotrophin Receptor Ectodomain Levels Across Clinically Diagnosed Alzheimer’s Disease, Late-Life Depression, and Healthy Aging: A Preliminary Cross-Sectional Study

This Supplementary Material contains Supplementary Tables S1–S4 and the legends for Supplementary Figures S1–S2. Supplementary Figures S1–S2 and Supplementary Data 1 are provided as separate files.

# Supplementary Table S1. Diagnostic-group-adjusted symptom and cognitive analyses

Each model used CSF p75NTR-ECD as the outcome and included the listed predictor, age, sex, BMI, and diagnostic group. Benjamini–Hochberg q values were calculated across the six predictor tests. HC3 results are sensitivity estimates.

| **Predictor** | **n** | **B** | **95% CI** | **p** | **FDR q** | **HC3 95% CI** | **HC3 p** | **HC3 q** |
| --- | --- | --- | --- | --- | --- | --- | --- | --- |
| HAM-D-21 total | 50 | -2.53 | -9.28 to 4.23 | 0.454 | 0.587 | -11.06 to 6.00 | 0.553 | 0.819 |
| HAM-D-21 sleep domain (items 4-6) | 50 | -23.03 | -46.92 to 0.86 | 0.058 | 0.117 | -49.77 to 3.71 | 0.090 | 0.179 |
| HAM-D-21 psychic anxiety domain (items 9-10) | 50 | -37.09 | -73.80 to -0.37 | 0.048 | 0.117 | -64.22 to -9.95 | 0.009 | 0.051 |
| MMSE total | 50 | 2.74 | -5.18 to 10.65 | 0.489 | 0.587 | -15.55 to 21.03 | 0.764 | 0.819 |
| MMSE orientation domain (items 1-2) | 50 | 4.86 | -14.23 to 23.95 | 0.610 | 0.610 | -37.63 to 47.34 | 0.819 | 0.819 |
| MMSE delayed recall (item 5) | 50 | 47.88 | 5.65 to 90.12 | 0.027 | 0.117 | 6.56 to 89.21 | 0.024 | 0.073 |

No predictor remained significant at FDR q < 0.05. Abbreviations: BMI, body mass index; CI, confidence interval; CSF, cerebrospinal fluid; FDR, false discovery rate; HAM-D-21, 21-item Hamilton Rating Scale for Depression; HC3, heteroscedasticity-consistent standard errors; MMSE, Mini-Mental State Examination; p75NTR-ECD, p75 neurotrophin receptor ectodomain.

# Supplementary Table S2. Sensitivity analyses for the primary group contrasts

All models adjusted for age, sex, and BMI. The plate, storage-duration, and freeze–thaw analyses added one further covariate in separate models.

| **Analysis** | **Contrast** | **n** | **B (pg/mL)** | **95% CI** | **p** | **Interpretation** |
| --- | --- | --- | --- | --- | --- | --- |
| Primary: all software-estimated values | HC vs AD | 50 | 131.2 | 42.8 to 219.6 | 0.005 | Consistent with primary direction |
| Primary: all software-estimated values | LLD vs AD | 50 | 154.6 | 68.9 to 240.3 | <0.001 | Consistent with primary direction |
| Sensitivity: values below 62.5 set to 62.5 pg/mL | HC vs AD | 50 | 123.9 | 38.7 to 209.0 | 0.005 | Consistent with primary direction |
| Sensitivity: values below 62.5 set to 62.5 pg/mL | LLD vs AD | 50 | 148.5 | 66.0 to 231.1 | <0.001 | Consistent with primary direction |
| Sensitivity: values below 62.5 excluded | HC vs AD | 47 | 78.3 | -2.8 to 159.5 | 0.058 | Attenuated; nominal p ≥ 0.05 |
| Sensitivity: values below 62.5 excluded | LLD vs AD | 47 | 124.5 | 44.8 to 204.2 | 0.003 | Consistent with primary direction |
| Sensitivity: additionally adjusted for plate | HC vs AD | 50 | 133.4 | 46.9 to 219.8 | 0.003 | Consistent with primary direction |
| Sensitivity: additionally adjusted for plate | LLD vs AD | 50 | 151.0 | 67.1 to 234.8 | <0.001 | Consistent with primary direction |
| Sensitivity: additionally adjusted for storage duration | HC vs AD | 50 | 131.6 | 35.6 to 227.6 | 0.008 | Consistent with primary direction |
| Sensitivity: additionally adjusted for storage duration | LLD vs AD | 50 | 154.0 | 51.9 to 256.1 | 0.004 | Consistent with primary direction |
| Sensitivity: additionally adjusted for freeze-thaw cycles | HC vs AD | 50 | 131.2 | 41.9 to 220.6 | 0.005 | Consistent with primary direction |
| Sensitivity: additionally adjusted for freeze-thaw cycles | LLD vs AD | 50 | 145.6 | 41.3 to 249.9 | 0.007 | Consistent with primary direction |

The three below-calibrator concentrations were 30.0 pg/mL (LLD), 41.2 pg/mL (AD), and 7.9 pg/mL (AD). They were retained in the primary analysis because they were the software-generated values in the original analytic dataset.

# Supplementary Table S3. Exploratory medication-dose analyses within LLD

Each model was a univariable linear regression with CSF p75NTR-ECD as the outcome. Benjamini–Hochberg q values were calculated across the three medication-dose tests.

| **Predictor** | **n** | **B** | **95% CI** | **p** | **FDR q** | **R²** |
| --- | --- | --- | --- | --- | --- | --- |
| IMI equivalents (mg/day) | 19 | -0.126 | -0.677 to 0.426 | 0.636 | 0.895 | 0.013 |
| CPZ equivalents (mg/day) | 19 | -0.064 | -1.077 to 0.949 | 0.895 | 0.895 | 0.001 |
| DME (mg/day) | 19 | 2.758 | -9.480 to 14.995 | 0.641 | 0.895 | 0.013 |

Abbreviations: CPZ, chlorpromazine; DME, diazepam milligram equivalents; FDR, false discovery rate; IMI, imipramine; LLD, late-life depression.

# Supplementary Table S4. Assay plate allocation and duplicate precision

| **Plate** | **HC** | **LLD** | **AD** | **Total** |
| --- | --- | --- | --- | --- |
| 1 | 7 | 7 | 4 | 18 |
| 2 | 6 | 6 | 4 | 16 |
| 3 | 6 | 6 | 4 | 16 |

All samples were measured in duplicate in one assay session. Median duplicate CV was 2.4% (range 0.1%–19.3%). Each plate contained all three diagnostic groups. Well positions were not randomized, and the operator was blinded to diagnostic group.

# Supplementary Figure Legends

Supplementary Figure S1. Associations between CSF p75NTR-ECD and psychotropic dose-equivalent measures within LLD. Crosses indicate individual participants. Lines show univariable least-squares fits for (A) IMI equivalents, (B) CPZ equivalents, and (C) DME. Nominal p values are displayed in the panels; the Benjamini–Hochberg FDR q value was 0.895 for each analysis. Abbreviations: CPZ, chlorpromazine; CSF, cerebrospinal fluid; DME, diazepam milligram equivalents; FDR, false discovery rate; IMI, imipramine; LLD, late-life depression; p75NTR-ECD, p75 neurotrophin receptor ectodomain.

Supplementary Figure S2. Q–Q plot of standardized residuals from the primary linear model. Open circles indicate standardized residuals, and the line indicates the theoretical normal reference. Residuals were approximately normal (Shapiro–Wilk W = 0.965, p = 0.146). Breusch–Pagan p = 0.501, White p = 0.272, and Durbin–Watson = 1.854 provided no evidence of major heteroscedasticity or autocorrelation.

# Supplementary Data 1 Legend

Supplementary Data 1. De-identified participant-level dataset and analysis outputs. The workbook includes de-identified variables used in the primary and exploratory analyses, a data dictionary, data-quality checks, assay plate and duplicate information, Table 1 recalculation, primary and sensitivity-model results, FDR-adjusted exploratory analyses, and analysis metadata. Direct identifiers, exact CSF collection dates, and free-text clinical notes were removed.
